# The Clinical and Pathological Characteristics of Refractory Pituitary Adenomas: A Single Center Experience

**DOI:** 10.3389/fonc.2022.846614

**Published:** 2022-03-16

**Authors:** Xiaohai Liu, Congxin Dai, Xinjie Bao, Kan Deng, Yong Yao, Ming Feng, Mingchu Li, Ge Chen, Renzhi Wang

**Affiliations:** ^1^Department of Neurosurgery, Xuanwu Hospital Capital Medical University, Beijing, China; ^2^Chinese Pituitary Specialists Congress, Beijing, China; ^3^Department of Neurosurgery, Tongren Hospital Capital Medical University, Beijing, China; ^4^Department of Neurosurgery, Peking Union Medical College Hospital, Chinese Academy of Medical Sciences and Peking Union Medical College, Beijing, China

**Keywords:** refractory pituitary adenoma, aggressive pituitary adenoma, tumor growth rate, Ki-67, EGFR

## Abstract

**Background:**

Most of pituitary adenomas (PAs) are slow-growing benign tumors which can be cured or controlled by conventional therapies, including surgery, medical treatment or radiotherapy. A small set of PAs, usually known as aggressive PAs or refractory PAs, present with more aggressive behavior and lead to poorer prognosis than classical PAs.

**Methods:**

We retrospectively analyzed the clinical and pathological characteristics of 44 patients who were diagnosed with refractory PAs by a multidisciplinary team (MDT). All the patients’ demographic characteristics, radiological findings, Knosp grade, treatment details and clinical outcomes were abstracted from the medical records. Additionally, 44 patients with nonrefractory PAs (NRPAs) matched for age and gender were selected to serve as the control group.

**Results:**

Despite using all combined treatments including surgery, radiotherapy and conventional medical treatments, all the refractory PAs showed tumor progression or hormone hypersecretion which caused increased morbidity and mortality and remained challenging to management. Compared with those of the non-refractory PAs, the tumor size, invasive rate and tumor growth rate (TGR) were significantly higher in the refractory PAs. TGR >2.2% per month may be considered as a preoperative indicator of refractoriness. The Ki-67 index in the refractory PAs were all ≥3%. EGFR, but not MMP2 or MMP9, was significantly overexpressed in refractory PAs compared with the corresponding levels in nonrefractory PAs.

**Conclusion:**

Refractory PAs are unresponsive to surgery, radiotherapy and conventional medical treatments with a poor prognosis. Moreover, a TGR ≥2.2% per month, Ki-67 index ≥3% and EGFR overexpression may be independent predictors of clinical refractoriness.

## Introduction

Pituitary adenomas (PAs), accounting for 15% of all intracranial neoplasms, are monoclonal benign tumors arising from adenohypophyseal cells (1). With the development of imaging technology, the prevalence of clinically evident PAs has recently been reported to be 1/1000 in a Belgian population and 0.776/1000 (of which 0.542/1000 were hormone secreting) in a region of the United Kingdom (2, 3). Interestingly, according to a meta-analysis of radiology and pathology autopsy reports, incidental findings of pituitary lesions were commonly found in almost 16.7% of the population, indicating that most PAs were nonfunctional tumors which stopped growing or grew very slowly and needed no intervention after tumorigenesis (4). In contrast, a small set of PAs, usually known as aggressive PAs or refractory PAs, exhibit radiologically invasive and unusually rapid tumor growth rates (TGRs) or clinically relevant tumor growth despite maximal treatment with standard therapies, including surgery, radiotherapy and conventional medical treatments (5–8). At the extreme end of the spectrum, PAs accompanied with noncontiguous craniospinal or distant metastasis are called pituitary carcinomas, which are exceedingly rare, comprising only 0.1–0.2% of all PAs (9). Although it is not clear why most PAs are slowly-growing benign tumors while others exhibit aggressive or even malignant behavior are still obscure, it is ultimately urgent to get an accurate identification and early treatment for these refractory PAs to improve the patient outcomes.

To differentiate refractory PAs with aggressive even malignant behavior from benign PAs for early diagnosis and intensive therapy, the 2004 World Health Organization (WHO) classification system categorized PAs as typical tumors, atypical tumors and pituitary carcinomas (10). PAs with Ki-67 labeling index of >3% and excessive staining with p53 were diagnosed as atypical PA. However, the clinical impact of atypical PA is controversial as many studies have shown that some atypical PAs do not grow in a clinically aggressive mode and remain quiescent for years during follow-up or after the initial total resection (11, 12). Due to its low predictive value, the category of atypical PA is abnegated in the updated 2017 WHO classification of PAs (13), which also insisted on the evaluation of tumor proliferation potential by mitotic count and Ki-67 labeling index. According to the European Society for Endocrinology (ESE) guidelines, the diagnosis of an aggressive PA should be considered in patients with a radiologically invasive tumor and unusually rapid TGR, or clinically relevant tumor growth despite optimal standard therapies, including surgery, radiotherapy and conventional medical treatment (14). However, this definition may seem obscure and ambiguous as it does not define what represents “clinically relevant tumor growth” or an “unusually rapid TGR”. Until now, no biomarkers have been used to predict the aggressiveness of PA and the outcome of patients.

During the last decade, we focused on the diagnosis and treatment of aggressive PAs and proposed a new category, “refractory PAs”, to define these adenomas which exhibit a distinctive disease course compared with that of benign PAs (7, 8, 15). Although the definition of “aggressive” and “refractory” overlaps with each other, the definition of refractory PA emphasizes the importance of the Ki-67 index, TGR and the patient’s response to the treatment from the standpoint of disease course. In order to optimize the definition of refractory PAs, here we investigated a series of 44 patients with refractory PAs to determine the clinical and pathological characteristics for early diagnosis and intensive intervention.

## Materials and Methods

### Patients

Between Jan. 2014 and Dec. 2016, 2021 patients with PA underwent transsphenoidal surgery (TSS) or craniotomy at Peking Union Medical College Hospital (PUCMH, Beijing, China). Forty-four patients who were diagnosed with refractory PAs according to our diagnostic criteria by a multidisciplinary team (MDT) that included endocrinologists, neurosurgeons, pathologists, neuroradiologists, and oncologists were enrolled in this study (6). In our previous studies, the most important characteristics of refractory PAs included refractoriness to standard therapies, including surgery, radiotherapy and conventional medical treatment; tumor infiltration of the adjacent structures based on either radiological images or intraoperative findings; a cut-off value of > 3% for Ki-67; increasing TGR > 2% per month; and tumor recurrence within 6 months after surgery (7, 8). All the patients’ demographic characteristics, radiological findings, Knosp grade, treatment details and clinical outcomes were abstracted from the medical records. Additionally, 44 patients with nonrefractory PAs (NRPAs) matched for age and gender were selected to serve as the control group. 40 cases in the NRPA group had been achieved total resection after the initial surgery, while 4 patients received subtotal resection and remained stable after external beam radiotherapy (EBRT). The study was approved by the Research Ethics Committee of PUMCH and all the patients provided their written informed consent for the research.

### Clinical and Radiological Evaluation

Visual field and visual acuity assessments were performed by neuroophthalmologist for all patients. Regrading functional tumors, endocrine-related complications related to tumor were also evaluated. Endocrine assessment was performed for all patients, and once every 6 months to 1 year during the follow-up. All patients underwent pituitary magnetic resonance imaging (MRI) scans. Then the cases were classified according to the Knosp classification system. TGRs were determined by calculating the velocity of tumor volume increases using a stereological method based on the Cavalieri principle in the patients with at least 2 thin-slice magnetic resonance images (MRI) (16).

### Immunohistochemical Staining

Immunohistochemical staining of all the PAs was performed on the paraffin blocks to test for adenohypophyseal hormones, pituitary transcription factors including Pit-1, SF-1, and T-pit and other biomarkers including Ki-67, low molecular weight cytokeratin (CAM 5.2), EGFR, p53, MMP-2 and MMP-9. In brief, sections with 5-μm thickness were stained using Ki-67 (Chemicon, USA) antibodies, p53 (ZSGB-BIO, China), CAM 5.2(ZSGB-BIO, China), MMP-2 and MMP-9 (Abcam, Cambridge, MA, USA), and EGFR (Cell Signaling Technology, Boston, MA). Sections incubated in phosphate-buffered saline alone served as negative controls. Three fields of view (400×) were randomly selected. The images were obtained under constant luminance without white balance. The integrated optical density (IOD) value was determined using Image-Pro Plus 6.0 software (Media Cybernetics, Inc., Silver Spring, MD, USA). The stained area was selected, and the other areas were hyalinized. The images were converted into grayscale images, and IOD values were calculated. A semiquantitative assessment of the immunohistochemical reactions for EGFR was used to score the staining as 0 (negative, IOD 0.1), 1+ (low, 0.1–0.4), 2+ (intermediate, 0.4–0.6), 3+ (high, 0.6–0.8), or 4+ (very high, >0.8). Quantification of Ki-67-labeled cells was assessed by counting more than 500 nuclei in four randomly selected high-power fields, excluding the nuclei of vascular components and hematological cells. Immunohistochemical protein expression was scored blindly by two observers using a conventional optical microscope (Olympus, Tokyo, Japan). p53 was considered positive if more than 10% of the nuclei stained densely. Negative staining was accepted when tumor cells were negative in areas with positive staining for endothelial and mesenchymal cells. Mitotic counts were performed by reviewing at least 20 high-power microscopic fields at × 400 magnification.

### Statistical Analysis

Statistical analysis was performed with SPSS 15.0 software (SPSS, Inc., Chicago, IL, USA). Comparisons of categorical variables were carried out by Chi-square or Fisher’s exact tests. Binary logistic regression was employed to analyze independent predicted variables of clinical refractoriness. When two-sided p values were ≤.05, the differences were considered statistically significant.

## Results

### Clinical Characteristics of Refractory PA

Among the 2021 patients with PA who underwent TSS or craniotomy at PUCMH, 44 refractory PAs were diagnosed, with an incidence of 2.2%. The clinical and immunohistochemical characteristics of 44 refractory PAs with integrated clinical archives and effective follow-ups are shown in **Table 1**. Among the 44 patients with refractory PAs, male patients accounted for 56.9% (25 cases), while female patients accounted for another 43.1% (19 cases). There were 824 male patients (40.7%) and 1197 female patients (59.3%) in the whole cohort. Fisher’s exact test revealed that the male sex distribution was significant (p < 0.05). The mean age at diagnosis in the refractory group was 46.6 years (range 21–80 years), while the mean age was 42.77 years (range 6–82 years) in the whole cohort, showing that patients with refractory PAs tended to be older than those with ordinary PAs (p < 0.05). Of the 44 patients with refractory PAs, 23 (52.3%) PAs were identified as clinically nonfunctional PAs, whereas 21 cases (47.7%) were functional PAs. Moreover, there were 4 gonadotroph adenomas (GAs), 5 lactotroph adenomas (LA), 6 somatotroph adenomas (SA), 8 corticotroph adenomas (CA), 7 Crooke’s cell adenomas (CCAs), 12 null cell adenomas (NCAs), 1 pit-1 positive plurihormonal adenoma (PPPA) and 1 acidophilic stem cell adenoma (ASCA) according to the 2017 WHO classification of PAs.

**Table 1:** Clinical and immunohistochemical characteristics of the 44 refractory PAs.

Pat. No.	Sex/Age	Tumor Type	SRS/EBRT	TSS/Craniotomy	Medical Therapy	Tumor Diameter(mm)	Ki67%	P53	Abundant Mitoses
1	M/59	SGLA	1/0	2/0	Bro	66.5	20	+	+
2	M/50	DGLA	1/0	2/2	Bro	57	20	+	+
3	F/60	SGLA	1/1	1/0	Bro	32	5	–	–
4	M/29	Acidophilic stem cell adenoma	1/0	0/1	Bro	57.5	3	–	–
5	M/60	SGLA	1/0	2/0	Bro	66	3	–	–
6	F/59	SGLA	1/0	1/0	Bro	38	20	–	+
7	F/67	NCA	0/1	1/0	–	58	3	–	–
8	F/30	NCA	0/1	1/0	–	42	3	–	–
9	F/21	NCA	0/1	1/1	–	50	5	–	+
10	F/56	GA	1/0	4/0	–	42	15	+	+
11	M/66	GA	0/1	1/0	–	128	10	+	–
12	F/55	NCA	0/1	1/0	–	56	3	–	–
13	M/80	NCA	1/0	1/0	–	45	3	–	–
14	M/32	SGSA	1/0	1/2	–	44	25	+	+
15	M/76	NCA	0/1	1/0	–	35	5	–	–
16	F/62	NCA	0/1	2/0	–	52	10	–	+
17	M/52	SGSA	1/0	3/0	–	18.4	5	–	–
18	M/40	NCA	1/0	3/0	–	52	3	+	+
19	M/24	SGSA	2/0	4/1	–	66	25	+	+
20	M/34	DGSA	0/1	3/1	–	72	5	–	–
21	F/26	NCA	0/1	1/0	–	14	3	+	+
22	F/55	NCA	2/0	3/1	–	69	3	–	–
23	M/66	NCA	1/0	1/0	–	53	3	–	–
24	F/27	NCA	0/1	1/0	–	82	3	+	–
25	M/25	PPPA	0/1	1/1	–	68	8	–	–
26	F/42	SGSA	1/0	1/1	–	32	3	–	+
27	F/29	SGSA	1/0	2/0	–	69	10	–	+
28	F/55	SGSA	1/0	1/0	Oct	12	3	–	–
29	F/42	DGSA	1/0	1/0	Oct	24	3	–	–
30	M/42	SGSA	1/0	1/0	Oct	16	3	–	–
31	M/76	SGSA	4/0	2/1	–	23	40	+	+
32	M/53	CCA	2/0	3/1	–	18	10	+	+
33	F/45	CCA	0/1	1/0	–	18	5	–	+
34	M/33	CCA	1/0	2/2	–	38	5	–	–
35	M/33	CCA	1/0	2/0	–	12	3	–	–
36	M/67	CCA	1/0	1/0	–	44	5	–	–
37	M/41	CCA	0/1	1/0	–	13	5	–	+
38	F/24	SGCA	2/0	1/0	–	12	3	–	–
39	M/39	CCA	1/0	1/0	–	26	3	–	–
40	M/30	SGCA	2/0	5/1	–	32	12	+	+
41	F/32	GA	2/0	0/5	–	64	25	+	+
42	F/63	SGCA	2/1	2/0	–	44	5	+	+
43	M/45	GA	1/0	1/2	–	39	5	–	–
44	M/50	SGCA	0/1	1/1	–	26	20	+	+

Bro, bromocriptine; CCA, Crooke’s cell adenoma; DGCA, Densely granulated corticotroph adenoma; DGLA, Densely granulated lactotroph adenoma; DGSA, Densely granulated somatotroph adenoma; GA, Gonadotroph adenoma; NCA, Null cell adenoma; Oct, Octreotide; PPPA, Plurihormonal PIT-1 positive adenoma; SGCA, Densely granulated corticotroph adenoma; SGLA, Sparsely granulated lactotroph adenoma; SGSA, Sparsely granulated somatotroph adenoma.

At diagnosis, the most common symptom among patients with refractory tumors was impaired vision or visual deficit (n = 27, 61.4%), followed by headache (n = 26, 59.1%), hypopituitarism (n = 21, 47.8%) and cavernous sinus syndrome (n = 10, 22.8%). All 5 patients with lactotroph adenoma and 1 patient with acidophilic stem cell adenoma had been treated and resistant to bromocriptine, and 3 in 6 GH tumors had been treated and exhibited resistance to octreotide. Regarding surgery (including TSS and craniotomy), all the 44 patients received at least one operation: 1 patient (2.3%) had undergone six operations, 2 patients (4.5%) had undergone five operations, 6 patients (13.6%) had undergone four operations, 5 patients (11.4%) had undergone three operations, 10 patients (22.7%) had undergone two operations, and 20 patients (45.5%) had undergone one operation. Regarding radiotherapy [including stereotactic radiosurgery (SRS) and external beam radiotherapy (EBRT)], all the 44 patients received at least one radiotherapy: 1 patient (2.3%) receiving four courses of SRS, 1 patient (2.3%) receiving three courses of therapy (two courses of SRS and one course of EBRT), 8 patients (18.2%) receiving two courses of therapy and the remaining 34 patients (77.3%) receiving one course of therapy at diagnosis. Despite the use of these combinations of treatments, all the refractory PAs showed tumor progression or hormone hypersecretion which caused increased morbidity and mortality and remained challenging to management.

For most the refractory PAs, follow-up MRIs revealed rapid growth of the residual tumor with invasion of the suprasellar cistern and cavernous sinuses which could not be resected either through TSS or craniotomy. To measure “rapid growth”, TGR was determined by calculating the velocity of tumor volume increases. TGR were calculated among the 28 refractory PAs with at least 2 thin-slice magnetic resonance images during follow-up, which varied from 2.2 to 12.4%/month. The mean TGR was 4.4%/month, which was significantly faster than that of nonrefractory PAs.

### Refractory Versus Nonrefractory PAs

**Figure 1:**
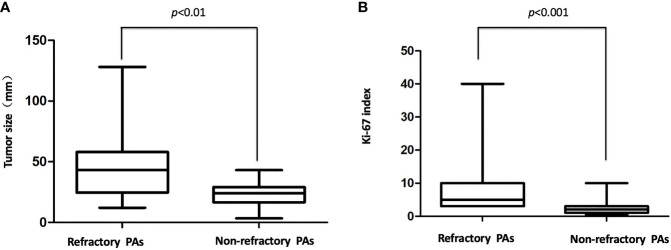
**(A)** Tumor size in the refractory and nonrefractory groups. There was a significant difference in the size of the refractory versus nonrefractory tumors (48.6 vs. 22.6 mm, p < 0.01 **(B)**, The Ki-67 index in the refractory and nonrefractory groups. There was a significant difference in the Ki-67 index of the refractory versus nonrefractory tumors (6.9% vs. 1.2%, p < 0.001).

To investigate the clinical characteristics of the patients with refractory tumors, 44 patients with nonrefractory PAs (NRPAs) matched for age and gender were selected to serve as the control group (**Table 2**). There were 11 gonadotroph adenomas (GAs), 2 lactotroph adenomas (LA), 12 somatotroph adenomas (SAs), 8 corticotroph adenomas (CAs), 1 Crooke’s cell adenomas (CCAs), 10 null cell adenomas (NCAs) according to the 2017 WHO classification of PAs. The diameters of the tumors in the refractory group ranged from 12.0 to 128.0 mm, with a mean size of 43.0 mm. In contrast, the diameters of the tumors in the nonrefractory group ranged from 3.3 to 43.0 mm, with a mean size of 22.6 mm. There was a significant difference in the size of the refractory versus nonrefractory tumors (48.6 vs. 22.6 mm, p < 0.01, **Figure 1A**). In addition, 24 of the 44 (54.5%) refractory PAs presented as a giant tumor (> 40 mm), while only 1 of the 44 (2.3%) PAs was a giant tumor in the NRPA (p < 0.01). In the refractory group, 37 tumors (84.1%) invaded the cavernous sinus, 23 tumors (52.3%) demonstrated suprasellar extension and 14 tumors (31.8%) extended into the clival region. Five tumors (four cases of Knosp grade 4 and one case of Knosp grade 3) in the nonrefractory group demonstrated cavernous extension both on MRI and during the operation and received near-total resection (NTR). There was no suprasellar extension or clival region invasion in the NRPA group. Compared with nonrefractory tumors, refractory tumors were more likely to be invasive (p < 0.01), suggesting that invasiveness was an independent predictor in the binary logistic regression. Thirty-nine patients in the NRPA group had been achieved total resection after the initial surgery, while 5 patients received NTR and remained stable after radiotherapy for a follow-up time of 31 months (range, 24-57 months). In contrast, 3 patients died of tumor progression and 1 patient died of tumor metastasis even with temozolomide therapy in the refractory group at a follow-up time of 20 months (range, 14–62 months).

**Table 2:** Clinical and immunohistochemical characteristics of the 44 non-refractory Pas.

Pat. No.	Sex/Age	Tumor Type	Knosp grade	Tumor Diameter	Ki67%	P53	Abundant Mitoses	Outcome
1	M/34	GA	2	24	1	–	–	CR
2	M/19	GA	2	21.9	10	+	+	CR
3	M/25	GA	2	24.8	2	–	–	CR
4	F/50	CCA	2	16	1	–	–	CR
5	M/63	NCA	4	38	1	–	–	SD after EBRT
6	F/35	NCA	3	29	1	+	+	CR
7	F/25	NCA	2	24	3	–	–	CR
8	F/53	NCA	2	20.3	2	+	+	CR
9	M/41	GA	3	31	3	–	–	CR
10	M/50	NCA	2	27.8	0.5	–	–	CR
11	M/22	GA	3	34	2	–	–	SD after EBRT
12	M/32	GA	3	29	3	–	–	CR
13	F/57	SGLA	1	18	1	–	–	CR
14	M/58	GA	4	34.2	3	–	–	SD after EBRT
15	F/43	SGSA	2	29	2	–	–	CR
16	M/30	DGSA	1	16	3	–	–	CR
17	F/36	DGSA	2	20	2	–	–	CR
18	F/67	GA	1	20	1	–	–	CR
19	M/53	DGSA	2	24	1	–	–	CR
20	F/40	DGLA	3	30	1	–	–	CR
21	M/33	SGSA	1	25	3	–	–	CR
22	M/72	DGSA	1	24	2	–	–	CR
23	F/51	NCA	1	22	4	–	+	CR
24	F/29	GA	2	27	10	+	–	CR
25	M/32	NCA	3	28	1	–	–	CR
26	F/39	NCA	2	21.2	1	–	–	CR
27	M/43	DGSA	2	25	1	–	–	CR
28	M/51	NCA	4	43	1	–	–	SD after EBRT
29	M/68	NCA	3	34	2	–	–	SD after EBRT
30	M/21	SGSA	1	11	1	–	–	CR
31	F/40	GA	3	36.7	2	–	–	CR
32	M/41	DGSA	2	22	2	–	–	CR
33	M/43	GA	2	25	2	–	–	CR
34	M/36	SGSA	1	19	5	+	–	CR
35	F/51	DGSA	1	15	3	+	–	CR
36	F/39	SGCA	1	3.3	2	–	–	CR
37	F/22	DGCA	1	13	5	–	–	CR
38	M/50	SGSA	3	30	1	–	–	CR
39	F/22	SGCA	1	10	1	–	–	CR
40	F/29	SGCA	1	18	2	–	–	CR
41	F/48	DGCA	1	11.1	1	–	–	CR
42	M/31	SGCA	1	6.2	3	–	–	CR
43	F/41	DGCA	1	10.2	2	–	–	CR
44	F/39	DGCA	1	3.5	1	–	–	CR

CCA, Crooke’s cell adenoma; CR, Complete remission; DGCA, Densely granulated corticotroph adenoma; DGLA, Densely granulated lactotroph adenoma; DGSA, Densely granulated somatotroph adenoma; EBRT, external beam radiotherapy; GA, Gonadotroph adenoma; NCA, Null cell adenoma; Oct, Octreotide; PPPA, Plurihormonal PIT-1 positive adenoma; SD, Stable disease; SGCA, Densely granulated corticotroph adenoma; SGLA, Sparsely granulated lactotroph adenoma; SGSA, Sparsely granulated somatotroph adenoma.

### Pathological Characteristics and Predictors for Clinical Refractoriness

**Figure 2:**
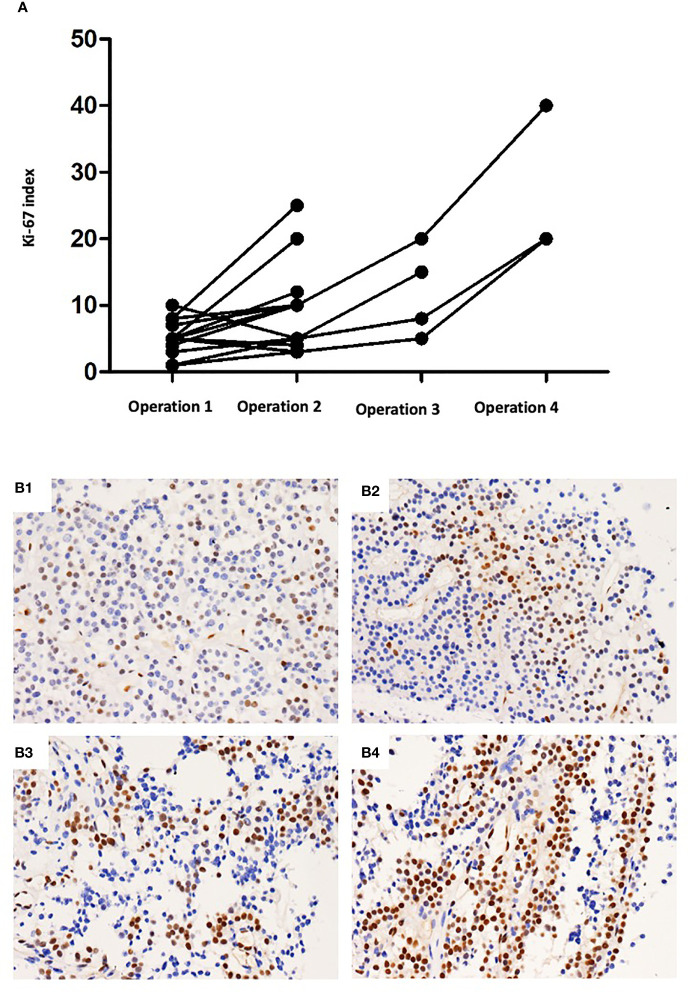
**(A)**The Ki-67 index increased over operations in 11 of 14 (78.6%) cases whose tissues were available from repeat surgeries. **(B)** Immunohistochemical staining of Ki-67 in one patient with refractory PAs who received four operations. **(B1)** 5% (×100 magnification). **(B2)** 10% (×100 magnification). **(B3)** 20% (×100 magnification). **(B4)** 40% (×100 magnification).

To investigate the pathological characteristics and identify the possible biomarkers predicting the refractoriness of PAs, immunohistochemical analysis was undertaken. In addition to biomarkers (such as Ki-67, mitotic index and p53 immunostaining) that were already used in the definition of atypical PAs investigated, but other biomarkers (such as MMPs and EGFR) were also assessed. For the histopathological examination, 13 of 44 (29.5%) refractory PAs displayed atypical features, including a Ki-67 labeling index above 3%, p53 staining and abundant mitosis, while none of the 44 nonrefractory PAs displayed atypical features (p < 0.0001). The Ki-67 index in the refractory group ranged from 3% to 40%, with a mean index of 8.6%, which was much higher than that among the nonrefractory tumors (1.2%, p < 0.001, **Figure 1B**). Binary logistic regression revealed that the Ki-67 index was an independent predictor of clinical refractoriness. Interestingly, the Ki-67 index increased over time in 12 of 14 (85.7%) cases whose tissues were available from repeat surgeries (**Figure 2**). The increasing Ki-67 index indicated progression of tumor malignancy with an increasing number of operations. In addition, p53 immunostaining was positive in 15 of 44 refractory PAs (34.1%), while only 1 of 44 nonrefractory PAs (2.3%) was positive for p53, indicating a significant difference (p < 0.001). Abundant mitosis/nuclear pleomorphism was seen in 20 of 44 (45.5%) refractory PAs (41.0%), while 2 of 44 nonrefractory PAs (4.5%) displayed abundant mitosis (p<0.001).

**Figure 3:**
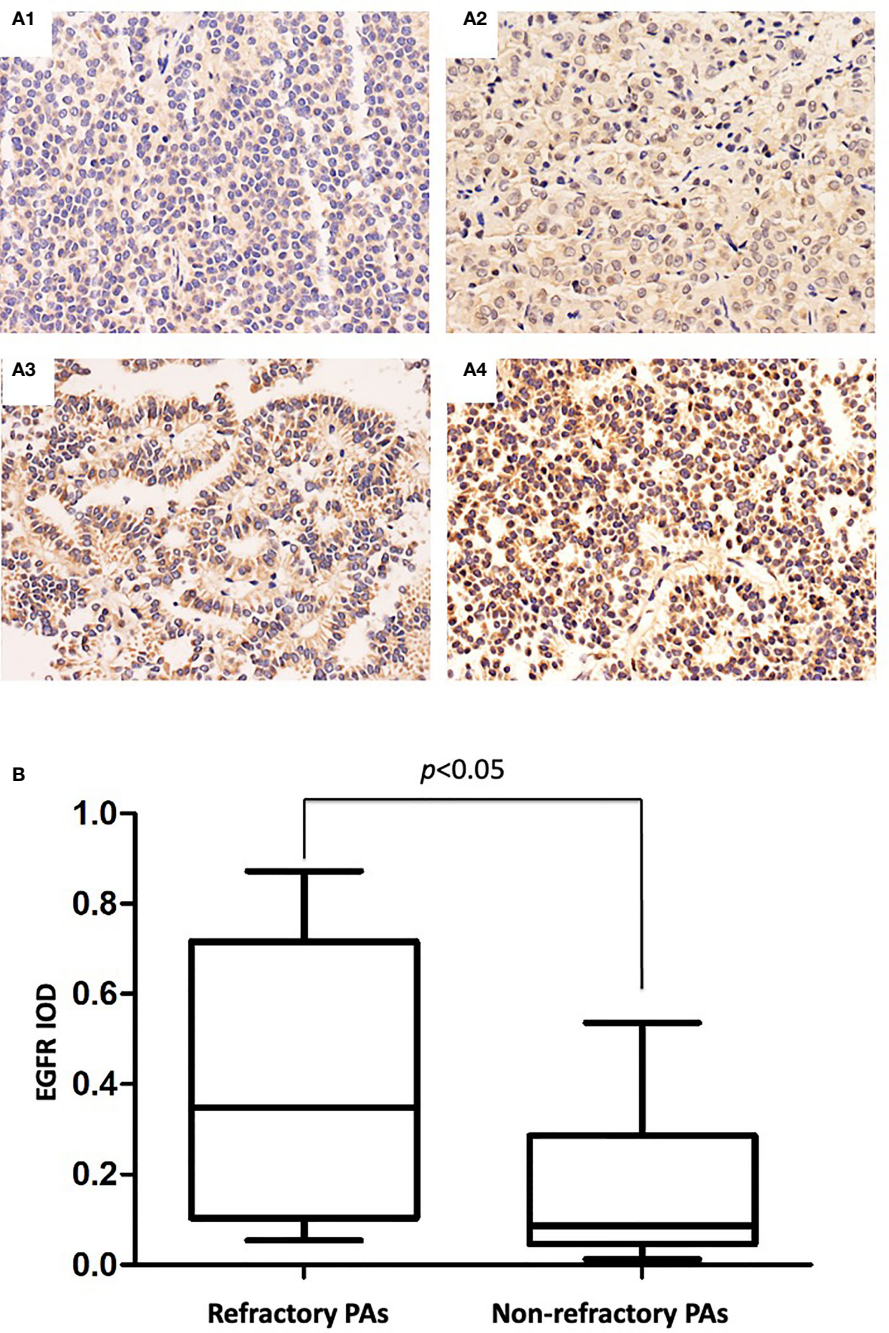
**(A)** Immunohistochemical staining of EGFR in PAs. **(A1)** IOD: 0.036 (×100 magnification). **(A2)** IOD: 0.192 (×100 magnification). **(A3)** IOD: 0.466 (×100 magnification). **(A4)** IOD: 0.842 (×100 magnification). **(B)**, EGFR mean IODs of refractory PAs and nonrefractory PAs. The mean EGFR IOD of the refractory PAs was significantly increased compared with that of the nonrefractory PAs (0.381 vs. 0.114, p < 0.05).

IHC staining showed strong EGFR immunoreactivity in 33 of 44 (75.0%) refractory PAs and 7 of 44 (15.9%) nonrefractory PAs. Representative images of EGFR immunohistochemical staining of refractory PAs and nonrefractory PAs are shown in **Figure 3A**. The mean IOD values of the refractory PA group and nonrefractory PA group were 0.381 and 0.114, respectively. Using an unpaired t test, we found that EGFR was significantly increased in refractory PAs compared with nonrefractory PAs (p < 0.01, **Figure 3B**). Binary logistic regression revealed that EGFR was an independent predictor of clinical refractoriness. There was no significant difference between refractory PAs and nonrefractory PAs regarding MMP2 or MMP9 (0.086 vs. 0.92, 0.122 vs. 0.114, both p >0.05).

## Discussion

The use of the terms “aggressive” and “refractory” as descriptors for some PAs is intended to distinguish PAs with malignant behavior and a poor prognosis from PAs having benign characteristics. According to the European Society of Endocrinology (ESE) published guidelines on the management of aggressive pituitary tumors and carcinomas, an aggressive pituitary tumor should be considered in patients with a radiologically invasive tumor and unusually rapid TGR, or clinically relevant tumor growth despite optimal standard therapies (surgery, radiotherapy and conventional medical treatments) (14). However, there is no general agreement on the definition of aggressive PAs regarding their specific clinical and pathological characteristics and the guideline did not determine what represented”clinically relevant tumor growth” or an “unusually TGR”. Therefore, the definition of aggressive PA relies mainly on subjective judgment of clinical characteristics but lacks objective diagnostic criteria and markers, leading to some confusion. Furthermore, “aggressive” and “invasive” are interpreted differently by different clinicians and are often used as interchangeable terms in the literature (17). In addition, there is a special situation in China where the Chinese words for “aggressive” and “invasive” are pronounced the same, which is more likely to lead to them being used interchangeably. Therefore, this was the initial reason for our proposal that a new term “refractory”, be used to define these PAs.

In our previous studies, the most important characteristics of refractory PAs include refractoriness to standard therapies, including surgery, radiotherapy and conventional medical treatment; tumor infiltration of the adjacent structures based on either radiological images or intraoperative findings; a cut-off value of > 3% for Ki-67; increasing TGR > 2% per month; and tumor recurrence within 6 months after surgery (7, 8). Although the definitions of aggressive and refractory PAs overlap with each other, we retrospectively analyzed 44 patients with refractory PAs and investigated their clinical and pathological characteristics, emphasizing the importance of the Ki-67 index, tumor growth velocity, and other features for the early diagnosis.

In the present case series, all the 44 patients with refractory PAs showed radiological invasiveness at diagnosis, and binary logistic regression demonstrated that invasiveness was an independent predictor of refractoriness in the binary logistic regression. Although most refractory PAs were invasive, a small number of the refractory PAs were noninvasive tumors, even microtumors at the time of first diagnosis (especially CD), indicating that invasiveness alone is insufficient to define refractoriness. At the early stage of a refractory PA, the tumor can be noninvasive. With the passage of time, the tumors may develop more invasive or aggressive characteristics, even progressing to malignancy (18, 19). Nevertheless, some PAs extending into the cavernous sinus and that were radiologically invasive were not aggressive. Cavernous sinus extension of PAs may be caused by the weakness of the medial wall of the cavernous sinus, not by the nature of the tumor (19). Additionally, some invasive tumors can be totally resected by one or multiple operations or controlled by radiotherapy (20). In our control group, a substantial number of invasive PAs achieved total resection through extended transsphenoidal surgery and showed no recurrence after a long follow-up time (**Figure 1**). Herein, invasiveness is not the always related to aggressiveness, and objective diagnostic criteria are needed, such as TGR and pathological features.

All the patients with refractory PAs received at least one or more operation. For those 20 patients (45.5%) who had undergone only one operation, the tumors invaded the cavernous sinus and encompassed the internal carotid artery, which could not be totally removed in the operation. All 20 patients who underwent one operation received one or more radiotherapies and showed tumor progression. After a mean follow-up of 44.2 months (range 6–88 months), 2 patients died of tumor progression and 1 patient died of tumor metastasis even with temozolomide therapy in the refractory group, suggesting not only that the clinical manifestation was more aggressive but also that the life expectancy of these PA patients was also markedly reduced (21, 22). Therefore, the most important characteristic of refractory PAs is that they are unresponsive to surgery, radiotherapy and conventional medical treatments with a poor prognosis.

The definition for aggressive does not provide an objective criterion for rapid growth, which easily leads to different judgments from different clinicians (23, 24). Here, the definition of refractory includes a TGR ≥2.2% per month, which can be used to determine rapid growth. Although more data are needed, it does provide an objective criterion for rapid growth.

Beyond the Ki-67 index which is already used in the definition of refractory adenomas, other biomarkers have also been investigated as potential biomarkers of refractoriness. Such biomarkers include matrix metalloproteinases (MMPs; including MMP2 and MMP9) which degrade extracellular matrix for enabling tumor invasion and epidermal growth factor receptor (EGFR), one subtype of ErbB receptors, which is expressed in PAs and regulates cell motility and adhesion, tumor invasion, angiogenesis and tumor cell proliferation (25). Previous studies showed that EGFR overexpression in transgenic mice driven by tissue-specific promoters induced PA tumorigenesis (25, 26), indicating its important role in pituitary tumorigenesis. In this study, we found that EGFR could also be used to assess refractory behavior. MMPs, particularly MMP2 and MMP9, are thought to play a central role in the proteolytic process of the extracellular matrix and basement membrane degradation, an essential step in these processes (27). However, neither MMP9 nor MMP2 could be used as an independent predictor of clinical refractoriness in our study.

## Conclusion

Refractory PAs are unresponsive to surgery, radiotherapy and conventional medical treatments with a poor prognosis. Moreover, a tumor growth rate ≥2.2% per month, Ki-67 index ≥3% and EGFR overexpression may be independent predictors of clinical refractoriness. Moreover, more cases and translational research are also needed to provide more insights into the definition of these refractory tumors.

## Data Availability Statement

The original contributions presented in the study are included in the article/supplementary material. Further inquiries can be directed to the corresponding author.

## Ethics Statement

The study was approved by the Research Ethics Committee of PUMCH and all the patients provided their written informed consent for the research. Written informed consent to participate in this study was provided by the participants’ legal guardian/next of kin. Written informed consent was obtained from the individual(s) for the publication of any potentially identifiable images or data included in this article.

## Author Contributions

All the authors participated in the design, collection and assembly of data, data analysis, and manuscript writing.

## Funding

The financial support for this study was provided by National Key R&D Program of China (grant number: 2021YFE0114300 to Renzhi Wang) and Beijing Hospitals Authority Youth Program (Code: QMS20210802 to Xiaohai Liu). The funding institutions had no role in the design of the study, data collection and analysis, decision to publish, or preparation of the manuscript.

## Conflict of Interest

The authors declare that the research was conducted in the absence of any commercial or financial relationships that could be construed as a potential conflict of interest.

## Publisher’s Note

All claims expressed in this article are solely those of the authors and do not necessarily represent those of their affiliated organizations, or those of the publisher, the editors and the reviewers. Any product that may be evaluated in this article, or claim that may be made by its manufacturer, is not guaranteed or endorsed by the publisher.
